# Supplementary material for: Changes in Growth, Ionic Status, Metabolites Content and Antioxidant Activity of Two Ferns Exposed to Shade, Full Sunlight, and Salinity
Source: Int J Mol Sci. 2022 Dec 24;24(1):296. doi: 10.3390/ijms24010296 (PMC9820618; doi:10.3390/ijms24010296)
Supplement: Supplementary file 1 [file ijms-24-00296-s001.zip › ijms-2021830-supplementary.pdf]

Supplementary Materials

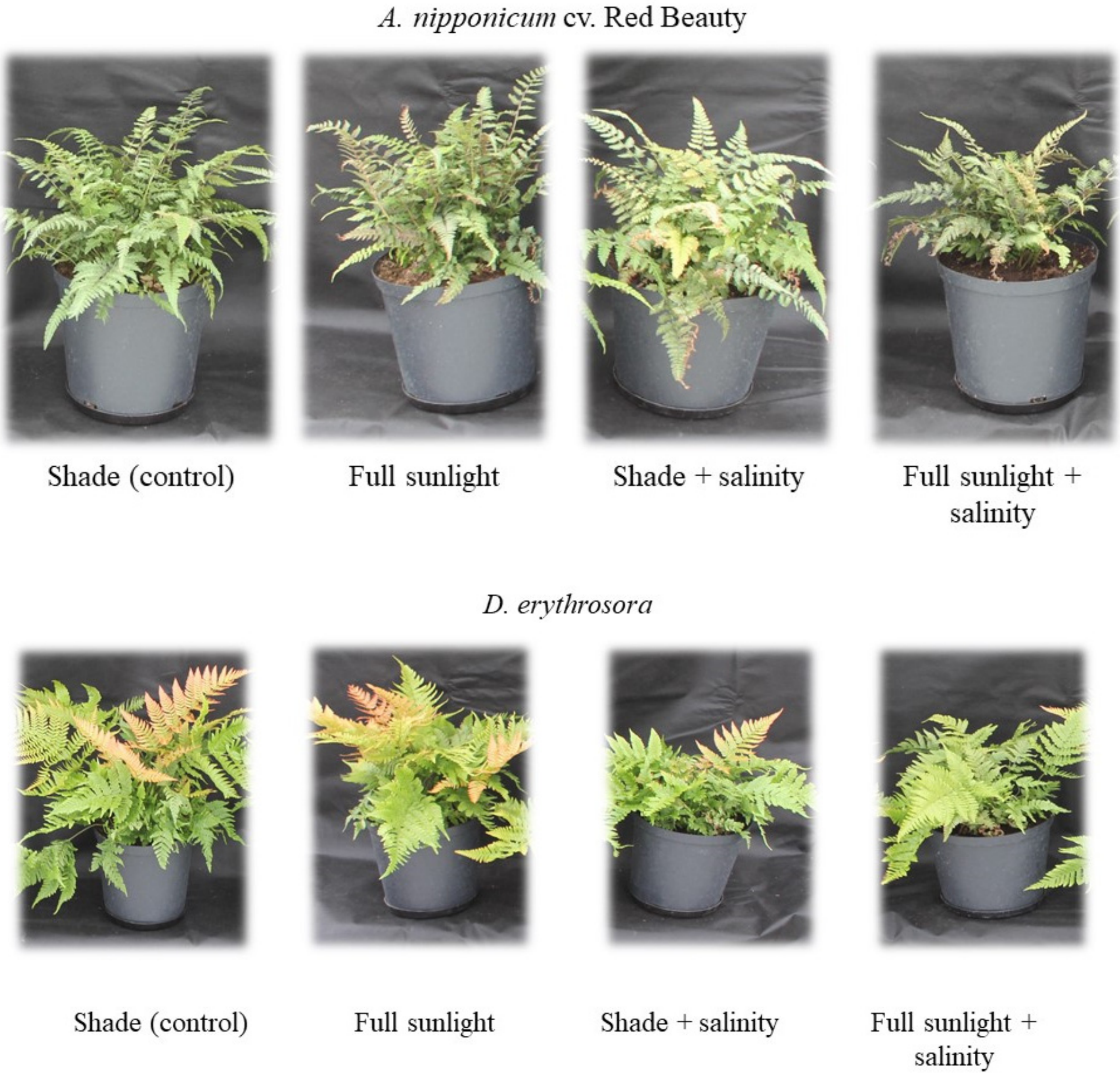

Figure S1. The appearance of plants grown in a polytunnel on the day of morphological measurements.

Table S1. Pearson correlation analysis of total free amino acids (TFAA), total phenolic content (TPC), total flavonoid content (TFC), and antioxidant activity (ABTS, DPPH, FRAP, Reducing Power – RP) of *A. nipponicum* cv. Red Beauty.

	TFAA	TPC	TFC	ABTS	DPPH	FRAP	RP
**TFAA**	1	0.71*	0.48	0.75*	0.86*	0.44	0.84*
**TPC**		1	0.34	0.45	0.60*	0.14	0.48
**TFC**			1	0.80*	0.27	0.03	0.55
**ABTS**				1	0.56	0.28	0.80*
**DPPH**					1	0.69*	0.86*
**FRAP**						1	0.74
**RP**							1

* Correlation is significant at the 0.05 level

Table S2. Pearson correlation analysis of total free amino acids (TFAA), total phenolic content (TPC), total flavonoid content (TFC), and antioxidant activity (ABTS, DPPH, FRAP, Reducing Power – RP) of *D. erythrosora*.

	TFAA	TPC	TFC	ABTS	DPPH	FRAP	RP
**TFAA**	1	0.26	0.57	0.13	0.08	0.35	0.69*
**TPC**		1	0.35	0.16	0.36	0.22	0.38
**TFC**			1	0.13	0.36	0.07	0.41
**ABTS**				1	0.80*	0.80*	0.38
**DPPH**					1	0.91*	0.46
**FRAP**						1	0.32
**RP**							1

* Correlation is significant at the 0.05 level
